# Susceptibility of Influenza Viruses to the Novel Cap-Dependent Endonuclease Inhibitor Baloxavir Marboxil

**DOI:** 10.3389/fmicb.2018.03026

**Published:** 2018-12-06

**Authors:** Emi Takashita, Hiroko Morita, Rie Ogawa, Kazuya Nakamura, Seiichiro Fujisaki, Masayuki Shirakura, Tomoko Kuwahara, Noriko Kishida, Shinji Watanabe, Takato Odagiri

**Affiliations:** Influenza Virus Research Center, National Institute of Infectious Diseases, Tokyo, Japan

**Keywords:** influenza virus, cap-dependent endonuclease inhibitor, baloxavir marboxil, baloxavir acid, S-033188, neuraminidase inhibitors, resistance

## Abstract

The novel cap-dependent endonuclease inhibitor baloxavir marboxil was approved for the treatment of influenza A and B virus infections in February 2018 in Japan. Because of the need to monitor influenza viruses for reduced susceptibility to this drug, we used two cell-based screening systems – a conventional plaque reduction assay and a focus reduction assay – to evaluate the susceptibility of influenza viruses to baloxavir. First, we generated a reference virus possessing an I38T substitution in the polymerase acidic subunit (PA), which is known to be associated with reduced susceptibility to baloxavir, and demonstrated the validity of our systems using this reference virus. We then determined the susceptibility of a panel of neuraminidase (NA) inhibitor-resistant viruses and their sensitive counterparts to baloxavir. No significant differences in baloxavir susceptibilities were found between the NA inhibitor-resistant and -sensitive viruses. We also examined seasonal influenza viruses isolated during the 2017–2018 influenza season in Japan and found that no currently circulating A(H1N1)pdm09, A(H3N2), or B viruses had significantly reduced susceptibility to baloxavir and none of the viruses possessed an amino acid substitution at PA residue 38. Use of a combination of methods to analyze antiviral susceptibility and detect amino acid substitutions is valuable for monitoring the emergence of baloxavir-resistant viruses.

## Introduction

In Japan, four neuraminidase (NA) inhibitors – oseltamivir, zanamivir, peramivir, and laninamivir – are approved for therapeutic or prophylactic treatment of influenza virus infection. In addition, favipiravir, a viral RNA-dependent RNA polymerase inhibitor, is approved and stockpiled for use against novel influenza virus infections where existing antivirals are ineffective. Because the surveillance of antiviral-resistant influenza viruses is important to protect public health and aid in clinical management, we have been conducting nationwide monitoring of the antiviral susceptibility of influenza viruses since 1999.

The novel cap-dependent endonuclease inhibitor baloxavir marboxil (S-033188) was approved on 23 February 2018 for the therapeutic treatment of influenza A and B virus infections and became available on 14 March 2018 in Japan. Treatment of influenza virus infections with this drug requires a single oral dose. The hydrolyzed active form of baloxavir marboxil (baloxavir acid; S-033447) inhibits the cap-dependent endonuclease of influenza A and B viruses (Koszalka et al., 2017). *In vitro* studies have revealed that an I38T substitution in the polymerase acidic subunit (PA) is associated with reduced susceptibility of influenza A(H1N1), A(H3N2), and B viruses to baloxavir (Noshi et al., 2018; Omoto et al., 2018). Furthermore, PA I38T and I38F substitutions emerged as a result of exposure to baloxavir marboxil in four (3.6%) of 112 A(H1N1)pdm09 viruses for which PA sequences were available in a Phase II clinical trial (Shishido et al., 2017; Omoto et al., 2018). In a Phase III clinical trial, PA I38T and I38M substitutions were detected after exposure to this drug in 9.7% of 370 A(H3N2) viruses (Hayden et al., 2018). Patients infected with the PA I38T or I38F mutant viruses exhibited prolonged virus shedding (Shishido et al., 2017) and the median time to alleviation of symptoms was longer in baloxavir recipients with I38T or I38M substitutions than in those without substitutions (Hayden et al., 2018). In a pediatric study, PA I38T and I38M substitutions emerged in 18(23.4%) of 77 A(H3N2) viruses (Omoto et al., 2018). Therefore, there is a need to develop convenient assays to monitor the baloxavir susceptibility of influenza viruses.

The susceptibility of influenza viruses to baloxavir has been determined by using a conventional plaque reduction assay (Shishido et al., 2017; Noshi et al., 2018; Omoto et al., 2018). However, the plaque reduction assay is not ideal for large-scale monitoring (Tisdale, 2000). Recently, a focus reduction assay was reported to be highly suitable for the surveillance of antiviral susceptibility in influenza viruses because it is sensitive, robust, and less laborious than conventional cell-based assays (Tilmanis et al., 2017). Here, we used two cell-based screening systems – a conventional plaque reduction assay and a focus reduction assay – to evaluate the baloxavir susceptibility of influenza viruses. We validated our systems by generating a reference influenza A virus possessing the PA I38T substitution. Using our approach, we examined the susceptibility of a panel of NA inhibitor-resistant viruses and their -sensitive counterparts to baloxavir. In addition, we subjected seasonal influenza viruses isolated during the 2017–2018 influenza season in Japan to the focus reduction assay to determine their baloxavir susceptibility and to genetic analysis to detect amino acid substitutions at residue 38 in PA.

## Materials and Methods

### Viruses

High-yield influenza A/Puerto Rico/8/34(H1N1) virus (A/PR/8/34) was generated by reverse genetics as previously described (Ping et al., 2015). A/PR/8/34 possessing the PA I38T substitution (A/PR/8/34-PA/I38T) was generated by using the QuikChange Lightning Site-Directed Mutagenesis Kit (Agilent). Eight pHH21-based RNA polymerase I plasmids for viral RNA synthesis and four protein-expressing plasmids to synthesize the viral replication complex were kindly provided by Dr. Yoshihiro Kawaoka (University of Wisconsin-Madison).

A panel of NA inhibitor-resistant viruses and their -sensitive counterparts were used in this study (Takashita et al., 2016; Table 2). A/Fukui/45/2004(H3N2), A/Fukui/20/2004(H3N2), plaque-purified B/Perth/211/2001-197E, and B/Perth/211/2001-197D were kindly provided by the isirv Antiviral Group^1^. Amino acid position numbering is A subtype and B type specific. Seasonal A(H1N1)pdm09, A(H3N2), and B viruses were isolated from patients during the 2017–2018 influenza season in Japan. Some of the patients were treated with baloxavir marboxil after specimen collection and therefore no clinical specimens were obtained from baloxavir-treated patients.

### Antiviral Compounds

Baloxavir acid was provided by Shionogi & Co., Ltd., or purchased from AbaChemScene or MedChemExpress. Oseltamivir carboxylate, peramivir, and zanamivir were purchased from Sequoia Research Products. Laninamivir was provided by Daiichi Sankyo Co., Ltd., Baloxavir acid was dissolved in dimethyl sulfoxide and oseltamivir carboxylate, peramivir, zanamivir, and laninamivir were dissolved in distilled water.

### NA Inhibition Assay

Cell-based assays are not suitable for monitoring the susceptibility of influenza viruses to NA inhibitors because amino acid substitutions in the hemagglutinin protein can affect the NA inhibitor susceptibility in the cell-based assays (Barnett et al., 2000; Tisdale, 2000). Therefore, the susceptibilities of the viruses to NA inhibitors were determined by using a fluorescence-based NA inhibition assay with the NA-Fluor influenza neuraminidase assay kit (Applied Biosystems). The results are expressed as the 50% inhibitory concentration (IC_50_). The IC_50_ values were calculated by using GraphPad Prism (GraphPad Software). To interpret NA inhibitor susceptibility, we used the World Health Organization (WHO) criteria, which are based on the fold-change in IC_50_ compared to the median for viruses from the same type/subtype/lineage showing normal inhibition (NI) (WHO, 2012). Reduced inhibition (RI) is defined as a 10- to 100-fold increase in IC_50_ for influenza A viruses, or a 5- to 50-fold increase in IC_50_ for influenza B viruses. Viruses showing highly reduced inhibition (HRI) are influenza A viruses with >100-fold increase in IC_50_ or influenza B viruses with >50-fold increase in IC_50_.

### Plaque Reduction Assay

The antiviral activity of baloxavir was determined by using a conventional plaque reduction assay as previously described (Takashita et al., 2016). Briefly, confluent monolayers of Madin-Darby canine kidney (MDCK) cells in 6-well plates were inoculated with 50 plaque-forming unit (PFU)/well of viruses. Virus adsorption was carried out for 1 h at 37°C and then the inoculum was removed. Then, 0.8% agarose in culture medium containing serial dilutions (0.025–2500 nM) of baloxavir acid was added to each well in triplicate. The cells were incubated for 3 days and the plaque numbers were counted. The susceptibilities of viruses to baloxavir were expressed as the IC_50_. The IC_50_ values were calculated by using GraphPad Prism.

### Focus Reduction Assay

The antiviral activity of baloxavir was also determined by using a focus reduction assay (Tilmanis et al., 2017) under Avicel overlays (Matrosovich et al., 2006). Confluent monolayers of MDCK cells in 96-well plates were inoculated with 100 μl of 1000 focus-forming unit (FFU)/well of viruses. Virus adsorption was carried out for 1 h at 37°C and then 100 μl of 1.2% Avicel RC-581 (FMC BioPolymer) in culture medium containing serial dilutions (0.025–2500 nM) of baloxavir acid was added to each well in triplicate. The cells were incubated for 24 h and then fixed with formalin. After the formalin was removed, the cells were immunostained with a mouse monoclonal antibody against influenza A or B virus nucleoprotein (Merck; MAB8251, MAB8661), followed by a horseradish peroxidase-labeled goat anti-mouse immunoglobulin (SeraCare) as previously described (Tilmanis et al., 2017). The infected cells were stained with TrueBlue Substrate (SeraCare) and then washed with distilled water. After drying, the number of FFU was quantified by using an ImmunoSpot S6 Analyzer, ImmunoCapture software, and BioSpot software (CTL) as previously described (Tilmanis et al., 2017; van Baalen et al., 2017). The IC_50_ values were calculated by using GraphPad Prism.

### Deep Sequencing

A cDNA library was prepared from viral RNA by using a NEBNext Ultra RNA Library Prep Kit for Illumina and NEBNext Singleplex Oligos for Illumina (New England Biolabs), followed by purification by using Agencourt AMPure XP (Beckman Coulter). The library was sequenced by using MiSeq Reagent Kits v2 with MiSeq (Illumina). Sequence reads were aligned to the reference sequences by using CLC Genomics Workbench 8 (CLC bio). All sequences are available from the EpiFlu database of the Global Initiative on Sharing All Influenza Data (GISAID).

### Statistical Analysis

Statistical analyses were performed using GraphPad Prism. Significant outliers were detected by using Grubb’s test. The Paired *t*-test and Fisher′s exact test were used to determine statistically significant differences between groups. *P-*values of <0.05 were considered statistically significant.

## Results

### Antiviral Susceptibilities of a Reference Influenza A Virus With a PA I38T Substitution

To examine whether the susceptibility of influenza viruses to baloxavir could be evaluated by using our cell-based screening systems (i.e., conventional plaque reduction and focus reduction assays), we conducted these assays using a reference influenza A(H1N1) virus with the PA I38T substitution that confers reduced susceptibility to baloxavir. The susceptibility of the virus to NA inhibitors was also analyzed by using a fluorescence NA inhibition assay.

The IC_50_ values of the virus to oseltamivir, peramivir, zanamivir, laninamivir, and baloxavir are shown in Table 1. Both the A/PR/8/34-PA/I38T mutant and the wild-type virus showed normal inhibition with all four NA inhibitors tested, whereas the A/PR/8/34-PA/I38T mutant virus exhibited 54- and 44-fold higher IC_50_ values to baloxavir in the plaque reduction and the focus reduction assay, respectively, compared with the wild-type virus, consistent with previous studies (Noshi et al., 2018; Omoto et al., 2018). Since the IC_50_ values obtained from cell-based assays are assay-specific, they cannot be compared directly with each other (Takashita et al., 2016). However, these results demonstrate that both cell-based screening systems are appropriate to evaluate the susceptibility of influenza viruses to baloxavir.

**Table 1:** Antiviral susceptibilities of a reference influenza A virus with a PA I38T substitution.

			IC_50_, nM (fold-change^a^)					
							Baloxavir	
Subtype	Virus	Amino acid substitution	Oseltamivir^b^	Peramivir^b^	Zanamivir^b^	Laninamivir^b^	Plaque reduction^c^	Focus reduction^d^
A(H1N1)	A/PR/8/34-PA/I38T	PA I38T	0.58 ± 0.16 (0.8)	0.09 ± 0.01 (1.0)	0.19 ± 0.01 (0.9)	0.24 ± 0.02 (1.4)	2.72 ± 0.89 (54)	7.40 ± 2.24 (44)
	A/PR/8/34	PA 38I (wild-type)	0.69 ± 0.10	0.09 ± 0.01	0.22 ± 0.01	0.17 ± 0.01	0.05 ± 0.02	0.17 ± 0.03

*IC_50_, 50% inhibitory concentration; PA, polymerase acidic subunit. ^a^Fold-change in IC_50_ values compared with the IC_50_ values of the wild-type virus. ^b^IC_50_ values were determined by use of a fluorescence neuraminidase inhibition assay. The values are presented as the mean ± SD of triplicate reactions. ^c^IC_50_ values were determined by use of a plaque reduction assay. The values are presented as the mean ± SD of triplicate reactions. ^d^IC_50_ values were determined by use of a focus reduction assay. The values are presented as the mean ± SD of triplicate reactions.*

### Baloxavir Susceptibilities of NA Inhibitor-Resistant Influenza Viruses

Next, we assessed the susceptibility of a panel of NA inhibitor-resistant viruses and their -sensitive counterparts to baloxavir by using our cell-based screening systems to confirm that NA substitutions did not affect the baloxavir susceptibilities. The influenza A(H1N1)pdm09-NA/H275Y, A(H3N2)-NA/E119V or -NA/R292K, and influenza B-NA/D197E mutant viruses exhibited HRI or RI against at least one of the four NA inhibitors, whereas no significant differences in baloxavir susceptibilities were found between the NA inhibitor-resistant and wild-type viruses by using either assay (Table 2); however, influenza B viruses showed higher IC_50_ values than influenza A viruses, as previously reported (Noshi et al., 2018; Omoto et al., 2018).

**Table 2:** Baloxavir susceptibilities of NA inhibitor-resistant influenza viruses.

			IC_50_, nM					
							Baloxavir (fold-change^c^)	
Type/subtype	Virus	Amino acid substitution^a^	Oseltamivir^b^	Peramivir^b^	Zanamivir^b^	Laninamivir^b^	Plaque reduction^d^	Focus reduction^e^
A(H1N1)pdm09	A/Chiba/1017/2009	NA H275Y	195.1 ± 26.2 HRI	28.0 ± 3.9 HRI	0.5 ± 0.1 NI	0.3 ± 0.1 NI	0.21 ± 0.02 (0.8)	0.53 ± 0.15 (1.0)
	A/Chiba/1016/2009	NA 275H (wild-type)	1.2 ± 0.7	0.1 ± 0.1	0.5 ± 0.1	0.2 ± 0.1	0.27 ± 0.09	0.50 ± 0.02
A(H3N2)	A/Fukui/45/2004	NA E119V	34.6 ± 8.9 RI	0.2 ± 0.1 NI	0.5 ± 0.1 NI	1.2 ± 0.1 NI	0.15 ± 0.02 (0.4)	0.62 ± 0.04 (0.5)
	A/Fukui/20/2004	NA 119E (wild-type)	0.1 ± 0.1	0.2 ± 0.1	0.5 ± 0.1	1.2 ± 0.1	0.42 ± 0.15	1.18 ± 0.49
	A/Kagoshima/2/2012	NA R292K	4816.0 ± 82.0 HRI	35.2 ± 2.6 HRI	10.9 ± 0.8 RI	3.9 ± 0.1 NI	0.22 ± 0.09 (1.5)	0.59 ± 0.29 (2.0)
	A/Kagoshima/4/2012	NA 292R (wild-type)	0.1 ± 0.1	0.1 ± 0.1	0.4 ± 0.1	0.5 ± 0.1	0.15 ± 0.06	0.29 ± 0.08
B	B/Perth/211/2001-197E	NA D197E	222.6 ± 14.4 RI	16.2 ± 6.0 RI	4.5 ± 4.0 RI	1.9 ± 0.1 NI	5.68 ± 1.00 (2.6)	5.63 ± 2.34 (1.0)
	B/Perth/211/2001-197D	NA 197D (wild-type)	13.9 ± 6.7	0.4 ± 0.1	0.6 ± 0.1	1.3 ± 0.3	2.20 ± 0.28	5.41 ± 1.99

*IC_50_, 50% inhibitory concentration; NA, neuraminidase. ^a^Amino acid position numbering is A subtype and B-type specific. ^b^IC50 values were determined by use of a fluorescence neuraminidase inhibition assay. The values are presented as the mean ± SD of triplicate reactions. HRI, highly reduced inhibition; RI, reduced inhibition; NI, normal inhibition. ^c^Fold-change in IC_50_ values compared with the IC_50_ values of the wild-type virus. ^d^IC_50_ values were determined by use of a plaque reduction assay. The values are presented as the mean ± SD of at least three reactions. ^e^IC_50_ values were determined by use of a focus reduction assay. The values are presented as the mean ± SD of triplicate reactions.*

### Baloxavir Susceptibilities of Seasonal Influenza Viruses Isolated During the 2017–2018 Influenza Season in Japan

**FIGURE 1:**
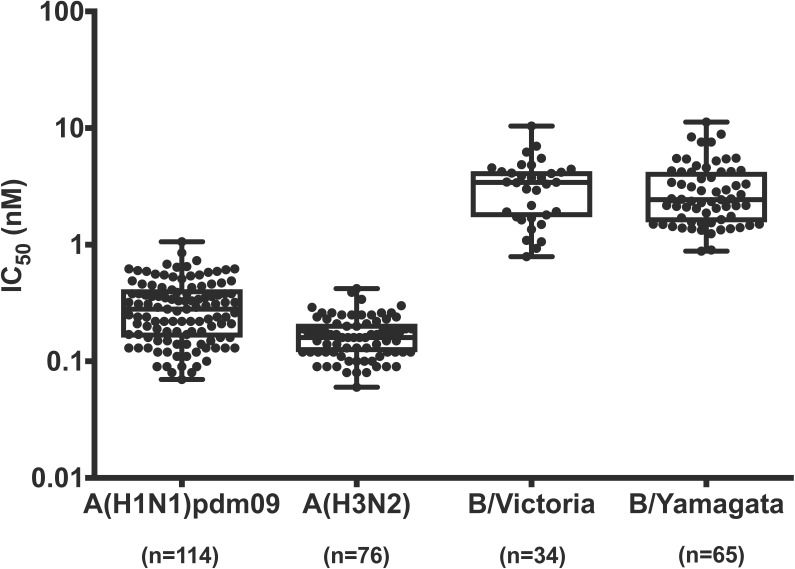
Baloxavir susceptibilities of seasonal influenza viruses isolated during the 2017–2018 influenza season in Japan. IC_50_ values were determined by use of a focus reduction assay. Box-and-whisker plots of IC_50_ values are shown.

To monitor the susceptibility of currently circulating influenza viruses to baloxavir, we examined the baloxavir susceptibility of seasonal influenza viruses [114 A(H1N1)pdm09, 76 A(H3N2), 34 B/Victoria-lineage, and 65 B/Yamagata-lineage viruses] isolated during the 2017–2018 influenza season in Japan by using the focus reduction assay (Figure 1). Influenza B viruses showed higher IC_50_ values than influenza A viruses as shown in Table 2. The median IC_50_ values of the viruses were 0.28 nM for A(H1N1)pdm09, 0.16 nM for A(H3N2), 3.42 nM for B/Victoria-lineage, and 2.43 nM for B/Yamagata-lineage viruses, respectively. The fold-changes in IC_50_ compared to the median for viruses from the same type/subtype/lineage ranged from 0.3 to 3.8 for A(H1N1)pdm09, from 0.4 to 2.6 for A(H3N2), from 0.2 to 3.0 for B/Victoria-lineage, and from 0.4 to 4.6 for B/Yamagata-lineage, respectively. These results indicate that none of the currently circulating viruses tested have significantly reduced susceptibility to baloxavir.

### Detection of Amino Acid Substitutions at Residue 38 in the PA Protein of Seasonal Influenza Viruses Isolated During the 2017–2018 Influenza Season in Japan

To detect amino acid substitutions at residue 38 in PA, we obtained whole-genome sequencing data by using the deep sequencing analysis. Seventy-four A(H1N1)pdm09, 149 A(H3N2), 25 B/Victoria-lineage, and 55 B/Yamagata-lineage viruses isolated during the 2017–2018 influenza season in Japan were analyzed (Supplementary Table 1). We found that none of the currently circulating viruses tested possessed the PA I38 substitution. Additionally, no other substitutions associated with reduced susceptibility to a prototypical PA endonuclease inhibitor were detected (Stevaert et al., 2013; Song et al., 2016).

## Discussion

Two influenza A(H1N1) viruses with reduced susceptibility to baloxavir were detected after seven or nine passages in Madin-Darby bovine kidney cells in the presence of this drug and the fold-change in EC_50_ values (i.e., the dose at which 50% of the maximum effect is produced) for each virus was 41 and 40, respectively (Noshi et al., 2018). These viruses possessed the I38T substitution in their PA protein. In the Phase II clinical trial of baloxavir efficacy in adults aged 20–64 years, PA I38T, I38F, and E23K substitutions were detected in A(H1N1)pdm09 viruses and a PA G548R substitution was detected in a B virus (Omoto et al., 2018). *In vitro* studies have revealed that influenza A(H1N1) viruses with the PA I38T, I38F, or E23K substitutions show 27.2, 10.6, and 4.7-fold higher EC_50_ values, respectively, whereas the PA G548R substitution in influenza B virus does not affect baloxavir susceptibility (Omoto et al., 2018). In the Phase III clinical trial in patients aged 12–64 years, PA I38T and I38M substitutions were detected in A(H3N2) viruses (Hayden et al., 2018). In the clinical trial of baloxavir efficacy in children aged 6 months to <12 years, the PA I38T and I38M substitutions were detected in 18 (23.4%) of 77 A(H3N2) viruses. Furthermore, A37T, E199G, N412D, V517A, and P632S substitutions were each detected in an A(H3N2) virus (Omoto et al., 2018). Influenza A(H3N2) viruses with the PA I38T, I38M, A37T, or E199G substitutions showed 56.6, 13.8, 8.1, and 4.5-fold higher EC_50_ values, respectively, whereas the baloxavir susceptibility was not affected by the PA N412D, V517A, or P632S substitution (Omoto et al., 2018). These results demonstrate that amino acid substitutions at residue 38 in PA are the major pathway for reduced susceptibility to baloxavir.

In this study, we used two cell-based screening systems – a conventional plaque reduction assay and a focus reduction assay – to evaluate the susceptibility of influenza viruses to baloxavir. Our data demonstrated that the reference virus possessing the PA I38T substitution showed 54- and 44-fold higher IC_50_ values to baloxavir in the plaque reduction and the focus reduction assay, respectively, indicating the validity of both systems for monitoring baloxavir susceptibility. Since the focus reduction assay can be used to analyze many viruses and is suitable for antiviral susceptibility monitoring (Tilmanis et al., 2017), we have initiated nationwide monitoring of the baloxavir susceptibility of currently circulating influenza viruses by using the focus reduction assay.

PA I38 is highly conserved among seasonal influenza A(H1N1)pdm09, A(H3N2), and B viruses (Jones et al., 2018). In our study, none of the currently circulating viruses tested possessed the PA I38T substitution. Omoto et al. (2018) reported that A(H1N1) and A(H3N2) viruses with the PA I38T, I38F, and I38M substitutions had impaired replication capability compared with the wild-type virus *in vitro*. In contrast, influenza B viruses with the PA I38T and I38M substitutions replicated comparable to the wild-type virus, although the PA I38F substitution in influenza B virus conferred impaired replication capability (Omoto et al., 2018). In the case of oseltamivir- and peramivir-resistant A(H1N1) and A(H1N1)pdm09 viruses with an NA H275Y substitution, some amino acid substitutions in the NA protein were able to compensate for the detrimental effect of the H275Y substitution on viral fitness (Bloom et al., 2010; Abed et al., 2011, 2014; Rameix-Welti et al., 2011; Bouvier et al., 2012; Butler et al., 2014). Thus, genetic analysis should also be conducted as part of nationwide monitoring to detect the acquisition of these permissive substitutions in the PA or other viral proteins.

*In vitro* studies of another new PA endonuclease inhibitor, RO-7, revealed that the PA I38T substitution was detected after serial passages of influenza A(H1N1) viruses in MDCK cells in the presence of RO-7 (Jones et al., 2018). Residue 38 is involved in the binding of baloxavir marboxil and RO-7 to the N-terminal domain of PA (Omoto et al., 2018). These findings suggest that the PA I38 substitution can confer multidrug resistance.

Previous studies reported PA amino acid substitutions associated with reduced susceptibility to L-742,001, a prototypical PA endonuclease inhibitor (Stevaert et al., 2013; Song et al., 2016). None of these substitutions were detected in our study, whereas some other PA substitutions were observed. Although their effect on baloxavir susceptibility remains unknown, we found that no influenza viruses tested had significantly reduced susceptibility to baloxavir.

In summary, a combination of phenotypic methods analyzing antiviral susceptibility and genotypic methods detecting amino acid substitutions is valuable for monitoring the emergence of baloxavir-resistant viruses.

## Author Contributions

ET, SW, and TO designed the analyses. ET, HM, RO, KN, SF, MS, TK, and NK analyzed and interpreted the data. ET drafted the article. SW and TO revised the article.

## Conflict of Interest Statement

The authors declare that the research was conducted in the absence of any commercial or financial relationships that could be construed as a potential conflict of interest.

## Footnotes

1 http://www.isirv.org/site/index.php/reference-panel

## References

[B1] Abed Y. Pizzorno A. Bouhy X. Boivin G. (2011). Role of permissive neuraminidase mutations in influenza A/Brisbane/59/2007-like (H1N1) viruses. *PLoS Pathog.* 7:e1002431. 10.1371/journal.ppat.1002431 22174688 PMC3234239

[B2] Abed Y. Pizzorno A. Bouhy X. Rheaume C. Boivin G. (2014). Impact of potential permissive neuraminidase mutations on viral fitness of the H275Y oseltamivir-resistant influenza A(H1N1)pdm09 virus in vitro, in mice and in ferrets. *J. Virol.* 88 1652–1658. 10.1128/JVI.02681-13 24257597 PMC3911590

[B3] Barnett J. M. Cadman A. Gor D. Dempsey M. Walters M. Candlin A. (2000). Zanamivir susceptibility monitoring and characterization of influenza virus clinical isolates obtained during phase II clinical efficacy studies. *Antimicrob. Agents Chemother.* 44 78–87. 10.1128/AAC.44.1.78-87.2000 10602727 PMC89632

[B4] Bloom J. D. Gong L. I. Baltimore D. (2010). Permissive secondary mutations enable the evolution of influenza oseltamivir resistance. *Science* 328 1272–1275. 10.1126/science.1187816 20522774 PMC2913718

[B5] Bouvier N. M. Rahmat S. Pica N. (2012). Enhanced mammalian transmissibility of seasonal influenza A/H1N1 viruses encoding an oseltamivir-resistant neuraminidase. *J. Virol.* 86 7268–7279. 10.1128/JVI.07242-12 22532693 PMC3416348

[B6] Butler J. Hooper K. A. Petrie S. Lee R. Maurer-Stroh S. Reh L. (2014). Estimating the fitness advantage conferred by permissive neuraminidase mutations in recent oseltamivir-resistant A(H1N1)pdm09 influenza viruses. *PLoS Pathog.* 10:e1004065. 10.1371/journal.ppat.1004065 24699865 PMC3974874

[B7] Hayden F. G. Sugaya N. Hirotsu N. Lee N. de Jong M. D. Hurt A. C. (2018). Baloxavir marboxil for uncomplicated influenza in adults and adolescents. *N. Engl. J. Med.* 379 913–923. 10.1056/NEJMoa1716197 30184455

[B8] Jones J. C. Kumar G. Barman S. Najera I. White S. W. Webby R. J. (2018). Identification of the I38T PA substitution as a resistance marker for next-generation influenza virus endonuclease inhibitors. *mBio* 9: e00430-18. 10.1128/mBio.00430-18 29691337 PMC5915737

[B9] Koszalka P. Tilmanis D. Hurt A. C. (2017). Influenza antivirals currently in late-phase clinical trial. *Influenza Other Respir Viruses* 11 240–246. 10.1111/irv.12446 28146320 PMC5410715

[B10] Matrosovich M. Matrosovich T. Garten W. Klenk H.-D. (2006). New low-viscosity overlay medium for viral plaque assays. *Virol. J.* 3:63. 10.1186/1743-422x-3-63 16945126 PMC1564390

[B11] Noshi T. Kitano M. Taniguchi K. Yamamoto A. Omoto S. Baba K. (2018). In vitro characterization of baloxavir acid, a first-in-class cap-dependent endonuclease inhibitor of the influenza virus polymerase PA subunit. *Antiviral Res.* 160 109–117. 10.1016/j.antiviral.2018.10.008 30316915

[B12] Omoto S. Speranzini V. Hashimoto T. Noshi T. Yamaguchi H. Kawai M. (2018). Characterization of influenza virus variants induced by treatment with the endonuclease inhibitor baloxavir marboxil. *Sci. Rep.* 8:9633. 10.1038/s41598-018-27890-4 29941893 PMC6018108

[B13] Ping J. Lopes T. J. Nidom C. A. Ghedin E. Macken C. A. Fitch A. (2015). Development of high-yield influenza A virus vaccine viruses. *Nat. Commun.* 6:8148. 10.1038/ncomms9148 26334134 PMC4569720

[B14] Rameix-Welti M. A. Munier S. Le Gal S. Cuvelier F. Agou F. Enouf V. (2011). Neuraminidase of 2007-2008 influenza A(H1N1) viruses shows increased affinity for sialic acids due to the D344N substitution. *Antivir. Ther.* 16 597–603. 10.3851/IMP1804 21685548

[B15] Shishido T. Kurihara N. Rokushima M. Omoto S. Noshi T. Naito A. (2017). “Genotypic and phenotypic monitoring of treatment-emergent resistance to S-033188, an influenza cap-dependent endonuclease inhibitor, in a phase 2, randomized, double-blind, placebo-controlled study in otherwise healthy adults with seasonal influenza,” in *Proceeding of the 5th ISIRV Antiviral Group Conference*, London.

[B16] Song M. S. Kumar G. Shadrick W. R. Zhou W. Jeevan T. Li Z. (2016). Identification and characterization of influenza variants resistant to a viral endonuclease inhibitor. *Proc. Natl. Acad. Sci. U.S.A.* 113 3669–3674. 10.1073/pnas.1519772113 26976575 PMC4822642

[B17] Stevaert A. Dallocchio R. Dessi A. Pala N. Rogolino D. Sechi M. (2013). Mutational analysis of the binding pockets of the diketo acid inhibitor L-742,001 in the influenza virus PA endonuclease. *J. Virol.* 87 10524–10538. 10.1128/JVI.00832-13 23824822 PMC3807387

[B18] Takashita E. Ejima M. Ogawa R. Fujisaki S. Neumann G. Furuta Y. (2016). Antiviral susceptibility of influenza viruses isolated from patients pre- and post-administration of favipiravir. *Antiviral Res.* 132 170–177. 10.1016/j.antiviral.2016.06.007 27321665

[B19] Tilmanis D. van Baalen C. Oh D. Y. Rossignol J. F. Hurt A. C. (2017). The susceptibility of circulating human influenza viruses to tizoxanide, the active metabolite of nitazoxanide. *Antiviral Res.* 147 142–148. 10.1016/j.antiviral.2017.10.002 28986103

[B20] Tisdale M. (2000). Monitoring of viral susceptibility: new challenges with the development of influenza NA inhibitors. *Rev. Med. Virol.* 10 45–55. 10.1002/(SICI)1099-1654(200001/02)10:1<45::AID-RMV265>3.0.CO;2-R 10654004

[B21] van Baalen C. A. Jeeninga R. E. Penders G. H. van Gent B. van Beek R. Koopmans M. P. (2017). ViroSpot microneutralization assay for antigenic characterization of human influenza viruses. *Vaccine* 35 46–52. 10.1016/j.vaccine.2016.11.060 27899226

[B22] WHO (2012). Meetings of the WHO working group on surveillance of influenza antiviral susceptibility - Geneva, November 2011 and June 2012. *Wkly. Epidemiol. Rec.* 87 369–374. 23061103

